# Analysis of Density, Roughness, and Accuracy of the Atomic Diffusion Additive Manufacturing (ADAM) Process for Metal Parts

**DOI:** 10.3390/ma12244122

**Published:** 2019-12-09

**Authors:** Manuela Galati, Paolo Minetola

**Affiliations:** Politecnico di Torino, Department of Management and Production Engineering (DIGEP) and Integrated Additive Manufacturing Center (IAM), 10129 Torino, Italy; manuela.galati@polito.it

**Keywords:** 17-4 PH, metal X, Markforged, dimensional accuracy, roughness, additive manufacturing

## Abstract

Atomic Diffusion Additive Manufacturing (ADAM) is a recent layer-wise process patented by Markforged for metals based on material extrusion. ADAM can be classified as an indirect additive manufacturing process in which a filament of metal powder encased in a plastic binder is used. After the fabrication of a green part, the plastic binder is removed by the post-treatments of washing and sintering (frittage). The aim of this work is to provide a preliminary characterisation of the ADAM process using Markforged Metal X, the unique system currently available on the market. Particularly, the density of printed 17-4 PH material is investigated, varying the layer thickness and the sample size. The dimensional accuracy of the ADAM process is evaluated using the ISO IT grades of a reference artefact. Due to the deposition strategy, the final density of the material results in being strongly dependent on the layer thickness and the size of the sample. The density of the material is low if compared to the material processed by powder bed AM processes. The superficial roughness is strongly dependent upon the layer thickness, but higher than that of other metal additive manufacturing processes because of the use of raw material in the filament form. The accuracy of the process achieves the IT13 grade that is comparable to that of traditional processes for the production of semi-finished metal parts.

## 1. Introduction

Additive manufacturing (AM) processes are revolutionizing the way engineers and designers can conceive and fabricate a product, thanks to the higher design freedom. Since the earliest Rapid Prototyping (RP) systems of the end of last century, nowadays AM machines are rapidly improving and developing toward efficient systems for the mass production of customized products. The absence of the need for a specific tool or die pushes the economic lot down to the single unit. Especially in the case of metal components, the interest of the industry is growing exponentially [1] because AM allows the production of fully dense near-net-shaped parts with complex structures made with excellent materials [2]. In fact, the main advantage of AM over conventional subtractive or formative methods is clearly illustrated by the greater product functionality that can be achieved by proper exploitation of the freedom in design [3,4].

The AM processes developed for metal components are mainly powder bed-based, in which an energy source is used to selectively melt the material. Due to the inefficiency and low power of the energy sources, the earliest metal AM machines were only indirect manufacturing systems because post-treatments were necessary to obtain a part with relatively good mechanical performance. In those earliest processes, the metallic powder was mixed with a polymeric binder.

**Figure 1:**
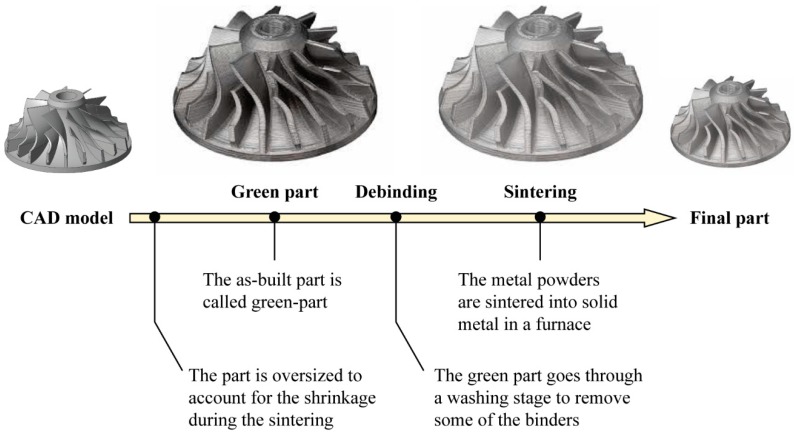
Workflow of the Bound Metal Deposition^TM^ (BMD) and the Atomic Diffusion Additive Manufacturing (ADAM).

The energy source of a laser beam was used to melt the binder which worked as an aggregator for the metal particles. The printed part, that included the binder and the metal materials, was named as the “green part”. The binder had to be removed by a post heat-treatment. The residual porosity, sometimes up to 40%, was replaced by means of infiltrations of copper or bronze [5,6]. Examples of such indirect AM processes are 3D printing or Selective Laser Sintering (SLS). Later, EOS developed a Direct Metal Laser Sintering (DMLS) system, in which the use of polymeric binder was avoided because the power of the implemented laser beam was high enough to sinter low melting alloys directly. In this case, the residual porosity to be filled up by the infiltration of a different material was lower and around 20% [5]. Recently, the development of energy sources with higher powers allowed engineers to overcome those first limitations and to unlock the technology potential. Today, a wider range of metals can be processed directly by AM to obtain dense metal parts without any additional post-processing operation. Laser or electron beam sources are used to fully melt the metallic powder [7], obtaining a fully dense (above 99.9%) part. In certain cases, the mechanical properties of additive manufactured parts are even better than the corresponding moulded material. Additionally, thanks to a controlled build chamber, materials with high melting point and/or high oxygen affinity, such as titanium alloys, can be processed more easily by AM than by conventional manufacturing [7]. However, AM powder bed-based processes present some design limitations related to the feasibility of enclosed cavities. In fact, after the construction, the unprocessed powder around the part needs to be removed by mechanical or manual operations. Enclosed cavities, that are not accessible externally, are therefore not allowed. This issue constrains the construction of lightweight parts that could also include enclosed lattice structures for providing stiffness, without a weight increase while supporting the loads. The use of powders also jeopardises the application of powder bed processes in extreme manufacturing scenarios, such as in weightlessness conditions [8], where powder management is not possible. To overcome these limitations, extrusion-based AM processes have been recently developed and launched onto the market. They draw inspiration from the wire-welding processes and from the Fused Deposition Modelling (FDM) technique, which is largely employed for layer-wise manufacturing of polymeric and composite parts. Compared to other AM processes, extrusion-based ones are more user-friendly and their equipment is less expensive. Furthermore, these systems are well suitable for the use of multi-material deposition. Electron Beam Additive Manufacturing (EBAM) is a direct AM process for large scale metal components, in which a metal wire is extruded while it is melted by an electron beam [7]. The main applications range from rapid prototypes and production parts to part repair. The American companies Desktop Metal Inc. and Markforged Inc. [9,10] recently launched two novel machines based on the combination of the Fused Deposition Modelling (FDM) process for polymers and Metal Injection Moulding (MIM) [11], a traditional process to obtain a close full density metal part with high complexity [12]. The process patented by Desktop Metal is called Bound Metal Deposition^TM^ (BMD), while Markforged Inc. named its process Atomic Diffusion Additive Manufacturing (ADAM). Both processes use a wire which is made of metal powders enclosed in a thermoplastic polymer that works as a binder for the metallic particles [13]. The mixture (metal powder particles and polymer) is stored in a cartridge on the top of the machine, and during the process, is fed into a unit in which the thermoplastic is softened to be easily extruded. BMD uses an ultrasonic vibrator to provide the energy necessary to bond the extruded material with the previously deposited one [13], while the Markforged Inc. system uses a simple heated extruder [10]. The softened material is accumulated and then pushed by a plunger trough a nozzle or extruder and deposited layer by layer on the build plate [14]. Like in the MIM process, the as-built part, also called the “green part”, is washed to remove the binder (debinding or washing operation), and then is sintered in a furnace that allows the densification of the material (sintering operation). Figure 1 depicts the schematic workflow of the process. The binder used in the Markforged Inc. system is completely debound thermally in the washing system before the sintering phase [14]. The binder used by Desktop Metal system is first debound by a solvent and then treated thermally [14]. Due to the presence of the binder and the sintering phase, the part needs to be scaled up and oversized to account for the shrinkage during post-processing. 

The Markforged MetalX machine has an in-situ inspection system based on a laser scanning sensor that monitors the dimensions of the part during the build [9]. Both systems by Markforged and Desktop Metal include a secondary extruder to deposit a layer of ceramic material on the interface between the supports and the parts during the construction. The ceramic layer is used to easily detach and remove the support structures at the end of the 3D printing phase. In this way, the 3D nesting of parts during the construction is also allowed. The powder used for the filament does not require any specification, thus also powders that have not been designed for AM can be used. Several materials been have advertised by the AM system manufacturers. Markforged proposes stainless steel (H13 tool steel and 17-4 PH), Inconel (625), Ti-6Al-4V, A-2 and D-2 for tooling, and aluminium alloys (6061 and 7075) [10]. However, only H13 tool steel and 17-4 PH are today industrialised and ready for production. Desktop Metal systems can process 17-4 and 316L stainless steel, 625 alloy, H13 tool steel, AISI 4140 and copper [13].

The aim of this work is to provide a characterization of the ADAM process using Markforged Metal X, the only commercial system currently sold on the market. The available material 17-4 PH, for which Markforged claims to be ready for industrial production, was considered. The density of the final parts is investigated by varying the layer thickness and the size of cubic samples. The surface roughness is measured on the different faces of the samples. The dimensional accuracy of the ADAM process is evaluated and defined by ISO IT grades using a reference artefact from the literature.

## 2. Materials and methods

### 2.1. Material and Equipment

In 2017 Markforged Inc. launched Metal X their own additive manufacturing (AM) machine for metals. The nominal composition of Markforged 17-4 PH material is reported in Table 1 [15].

The building volume of the machine is 300 × 220 × 180 mm^3^, but the maximum part size that can be built is 250 × 183 × 150 mm^3^. At the start of each job, a vacuum-sealed sheet is placed on the top of the build platform to facilitate the adhesion of the part during the construction and its detachment at the end of the process. The building platform and levelling system are designed to carry a maximum load of 10 kg. The chamber and the building platform are heated during the process.

Markforged WASH-1 was used as a debinding system. The washing vat is 356 × 254 × 203 mm^3^ and the washing fluid was Opteon Sion.

Markforged Sinter-1 furnace was used for the sintering phase. It can achieve a temperature up to 1300 °C and can work in an inert atmosphere using Argon and Nitrogen. The chamber has a cylindrical volume with a diameter of 141 mm and a length of 305 mm.

**Figure 2:**
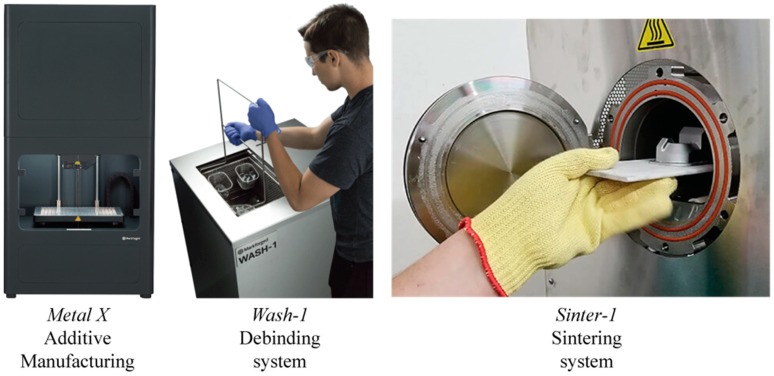
Markforged equipment for the Atomic Diffusion Additive Manufacturing (ADAM) process workflow (Credit: Markforged Inc.).

Figure 2 depicts the three systems used to complete the Atomic Diffusion Additive Manufacturing (ADAM) process workflow.

The part size and the whole ADAM process parameters, including the support structure, are designed automatically by the proprietary software (d5dda4f released in July 2019) named Eiger. Eiger is a CAM software that manages the whole process from the design to the sintering phase. The software is closed to the user. Therefore, the process parameters are not known, and cannot be varied by the user, except for the layer thickness that represents the resolution of the machine. Today, the layer thickness can be set at 0.050 mm or 0.125 mm. However, this 0.050 mm layer height is an alpha feature, and is still under development. Once the layer thickness is set, Markforged Eiger software (d5dda4f released in July 2019) calculates the volume expansion to account for the shrinkage of the part during the post-printing operations. Then it provides the final geometry to be printed and the minimum time required for the debinding of the polymer (washing time). Markforged Eiger software also defines the thermal heat treatments for the subsequent sintering step.

**Figure 3:**
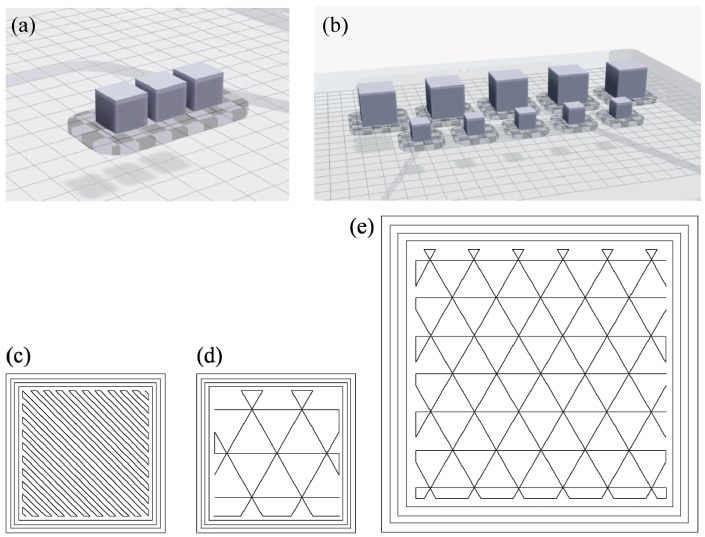
Build jobs for the evaluation of the material density. (**a**) Job A: production of three cubes with nominal dimension 10 × 10 × 10 mm^3^ and 0.050 mm layer thickness (set FD); (**b**) Job 2: production of five cubes with nominal dimension 10 × 10 × 10 mm^3^ and 0.125 mm layer thickness (set TC1) and five cubes with nominal dimension 20 × 20 × 20 mm^3^ and 0.125 mm layer thickness (set TC2); Scaled section and infill strategy for the samples of sets FD (**c**), TC1 (**d**) and TC2 (**e**).

The deposition strategy includes one outer contour and three inner ones (Figure 3c–e). Like in standard Fused Deposition Modelling (FDM) systems, the bottom and the top layers are printed with full density, while the strategy used for the other layers depends on the layer thickness. For the layer thickness of 0.050 mm a full-density (FD) strategy is used (Figure 3c), while a closed triangular cell (TC) path is followed to infill the section for the higher layer thickness of 0.125 mm (Figure 3d,e).

### 2.2. Density Evaluation

**Figure 4:**
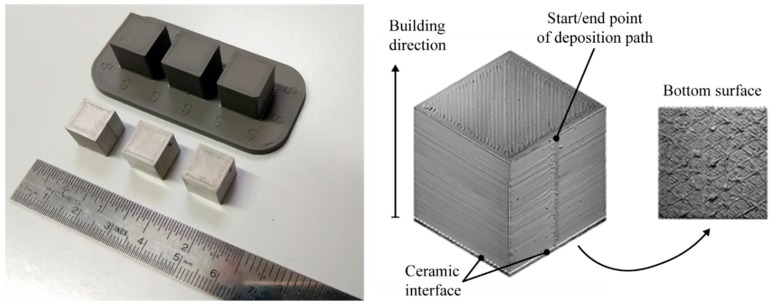
Cubic samples with nominal dimension 10 × 10 × 10 mm^3^ before and after the sintering phase (**left**). Typical surface texture of the top, bottom and lateral surfaces of cubes produced by the ADAM process (**right**).

Cubic samples measuring 10 × 10 × 10 mm^3^ were produced using both layer thicknesses of 0.050 mm and 0.125 mm to evaluate the final density of the manufactured parts. A first set FD of samples included three replicas of the cube that were printed at the highest resolution, i.e., with a layer thickness of 0.050 mm. A second set TC1 of five cubes was fabricated with the coarser resolution and 0.125 mm layer. An additional set (TC2) of five cubes with dimensions equal to 20 × 20 × 20 mm^3^ was produced using the same layer thickness of 0.125 for evaluating the effect of the sample size. Figure 3 shows the build jobs in Eiger software and the infill strategies for the internal cross-section of the samples. The cubes have been scaled and oversized automatically by the software to compensate for the final shrinkage of the part due to the sintering phase. The oversized cube has an edge length of 12 mm for the samples with a nominal dimension of 10 mm. For the bigger samples the oversized dimension was 23.9 mm for the nominal edge length of 20 mm. This means that Markforged estimated the post-processing shrinkage of the green part equal to 20%, which is the scaling factor implemented into Eiger software for a linear dimension. The estimated volume shrinkage is therefore 72.8%. Table 2 resumes the production data for the cubic samples. Job A is the build job that includes the set FD, while Job B includes the ten cubes from sets TC1 and TC2. Under each sample a raft was added to improve the adhesion of the part to the building platform, with the ceramic layer at the interface between the raft and the cubes (Figure 4).

Figure 4 shows the cubes of set FD before and after the sintering phase. On the right of Figure 4 microscope imagines show the detail of the top, lateral and bottom surfaces as they appear on the real as-built sample. In this figure, superficial defects and deposition strategies of the metallic filament can be recognised. One of the four side faces of the cube clearly shows a vertical line at the point where the filament deposition path starts and ends at each layer. The bottom surface shows the deposition strategy that is used to ease the part detachment from the raft. 

After the production, the three dimensions of the as-built cubic samples or green parts have been measured with a micrometer. Each measurement was replicated three times. The average values of the length (*l*), width (*w*) and height (*h*) of the cubes with their deviation range are reported in Table 3. In particular, the height (*h*) corresponds to the dimension of the samples in the build direction and layer growth.

After the washing phase, the cubes were sintered in one single stage. After sintering, the measurements of the sample dimensions were repeated (Table 3).

The density of the samples was measured by means of a hydrostatic balance using distilled water at 25 °C [16,17]. Unlike other methods, the hydrostatic balance allows calculating the effective density, considering the actual volume of the sample. First of all, the weight of the sample is measured in air. A subsequent immersion in the fluid allows an evaluation of the actual volume of the sample. The actual volume includes the enclosed porosities into the sample. An additional weighting of the sample after the immersion allows differentiating the enclosed porosities from the superficial pores that bring the fluid inside the sample from the external surface. The amount of fluid that remains trapped in these pores increases the weight of the sample. The superficial porosities are called open porosities, and could be due to the surface roughness or the presence of superficial cracks. Therefore, they need to be excluded from the density calculation. The following procedure has been adopted [16,18]:

(1) Weighing of the cube (weight in air). The measurement has been replicated three times;
(2) Positioning of the sample on the hydrostatic suspension, immersion in distilled water at 25 °C and reading of the weight (weight in water). The sample is immersed in the fluid by positioning the surface that was attached to the building platform or raft as the bottom surface;
(3) The sample is removed from the water and then it is dried externally and weighed again (wet weight).
(4) Previous steps 2 and 3 are repeated three times to get measurement replicas.

The effective density considers both the open and the enclosed porosities [19,20], and is calculated as follows:

$$Effective\;density=\;\frac{Water^{\prime }s\;density\times weight\;in\;air}{wet\;weight-weight\;in\;water}$$

where the water’s density at 25 °C is assumed to be equal to 997 kg/m^3^. The relative density is the ratio between the effective density and the nominal density of the 17-4 PH which was assumed to be equal to 7750 kg/m^3^ [21].

### 2.3. Evaluation of the Surface Roughness

The surface roughness profiles were measured by an RTP-80 profilometer by Metrology Systems with a TL90 drive unit. A cut-off length of 0.8 mm and a sampling length equal to five cut-off lengths were used [22]. The profiles were acquired in the centre of the surface by positioning the drive unit perpendicular to the surface. For each surface, three measures were collected. The Ra value has been used as a descriptor for the surface roughness.

### 2.4. Methodology for Evaluating the Dimensional Accuracy

**Figure 5:**
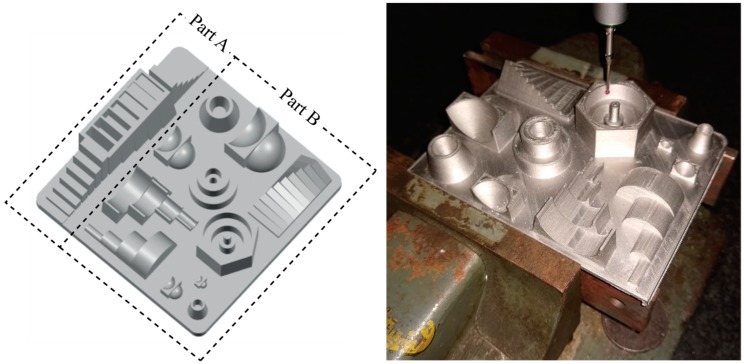
Geometry of the reference artefact [16] for the evaluation of ADAM process accuracy (Overall dimensions: 110 × 110 × 33 mm^3^) and dimensional inspection of Part B with a coordinate measuring machine (CMM).

The accuracy analysis aims to evaluate the geometrical and dimensional accuracy that can be obtained from the ADAM process. The analysis was carried out using the reference part proposed by Minetola et al. [23], which was already adopted for the evaluation of the accuracy of several 3D printing systems based on the extrusion of a polymeric filament [24,25]. The reference part comprises several simple geometries of different dimension to characterise the accuracy for the first eight ranges of the ISO basic size [26]: 1–3 mm, 3–6 mm, 6–10 mm, 10–18 mm, 18–30 mm, 30–50 mm, 50–80 mm and 80–120 mm. Figure 5 shows the geometry of the artefact which can be downloaded for free as an STL file from the GrabCAD library [27]. Dimensional and geometrical tolerances, including form errors, are evaluated for the convex and concave features of the artefact accordingly to the GD&T system.

Due to the need to scale up the dimensions to account for the shrinkage during the ADAM sintering phase and the limitation of the chamber size of the SINTER-1 furnace, the artefact was split into two parts “Part A” and “Part B” (Figure 5). Those parts were manufactured and post-processed separately to fit in the furnace. Table 4 resumes the production data for the artefact replica split into Part A and Part B.

After the production and the washing, the two parts of the replica were sintered in the SINTER1 furnace using the standard cycle.

The evaluation of the dimensional accuracy of the replica was carried out according to the ISO 286-1:1988 guideline [28]. For each range of the ISO basic size, the dimensional accuracy of the ADAM process was evaluated considering the achieved IT grade of the artefact replica. In particular, the IT grade was defined assuming the maximum dimensional error as the number of unit tolerance n corresponding to the 95th percentile of the distribution of the number of unit tolerance *n_j_* for the generic *j*^th^ dimension, in which *n_j_* is calculated as follows:

$$n_{j}=\;\frac{1000\;\left|D_{jn}-D_{jm}\right|}{i}$$

where here *D_jn_* is the nominal dimension, *D_jm_* is the actual dimension of the feature and *i* is the tolerance factor that varies among different ranges of the ISO basic size (Table 5). The actual dimension is assumed as the average of three replications of the measurement of the single geometric feature of the replica. The measurements were carried out by a coordinate measuring machine (CMM) by Brown and Sharpe (Figure 5). The CMM model is the GLOBAL Image 07.07.07 that has a declared Maximum Permissible Error (MPE_E_) of 1.5 + L/333μm according to ISO-10360/2 [29], where L is the measured length. Table 6 shows the classification of the dimensional quality as the ISO IT grades depend on the n value. 

## 3. Results and Discussion

### 3.1. Density Evaluation

The measurement of the cubic samples before the sintering phase (Table 1) shows an isotropic scale factor that does not depend on the layer thickness. The comparison between the dimensions of the cubes before and after the sintering phase shows that the samples which were built with a lower layer thickness (set FD) have a higher shrinkage factor if compared to the samples which were built with a layer thickness of 0.125 mm (sets TC1 e TC2). In addition, the height (*h*) is always larger than the width (*w*) and length (*l*) of the sintered cubes. Therefore, lower material shrinkage occurs along the build direction. For this reason, the values of the height have a lower standard deviation, meaning that the ADAM process can be better controlled.

However, the layer thickness appears to have a significant effect as well. The cubes from the sets FD and TC1 which are nominally equal show differences in dimensions. The cross section of the FD cubes is smaller than that of the TC1 samples. The highest deviation from the nominal dimensions was observed for the samples of set TC2, which is lower than 4%.

The volumetric shrinkage of FD cubes is around the 72.6% that well agrees with the expected theoretical value (72.8%). The values for TC1 and TC2 cubes are around 70.1% and 51.7%, respectively. The differences could be symptomatic of a diverse heat transfer according to the dimensions and filling strategy of the samples.

Table 7 resumes the averages of the replicated measurements of sample weight which were carried out according to the procedure described in the previous Section 3.

The cubes of set P show a relative density higher than those of sets TC1 and TC2. Therefore, the different strategies used for the infill at different layer thicknesses caused a different residual porosity. The density of the FD samples which were built with full-density settings is around 90%, which is far below the value provided by Markforged Inc. on official datasheets [15]. This measured density is also in discordance with the values provided by Condruz et al. [30], who analysed the effect of heat treatment on ADAM printed parts. However, Condruz et al. evaluated the porosity by image processing through the analysis of one single cross-section of their sample [30]. On the contrary, in this work the density is evaluated on the whole volume, thanks to the use of the hydrostatic balance.

The internal porosity of the cubes produced with a layer thickness equal to 0.125 mm is an index of the percentage of infill of the section. The comparison between the two values for the sets TC1 and TC2 shows a significant effect of the dimension of the samples. In fact, the smaller cubes have a higher relative density than the bigger ones.

For all samples, a significant effect on the actual density is given by the open porosities. These porosities might be caused by the texture of the bottom surface that is the one attached to the building platform. To verify this assumption, the bottom surface of the samples was polished and then the density re-calculated. Table 8 resumes the new results of the density measurement.

After the polishing operation, the open porosities are close to zero in most of cases. This result confirmed the previous assumption about the texture of the bottom surfaces. Therefore, with the exception of the bottom faces of the cubes, the other surfaces appeared to be well defined in terms of density with no external pores. This means that the layers of the samples are well welded to each other, and there is a good adhesion between them.

The cubes from the FD set that were built at the highest resolution (0.050 mm) for the machine show residual internal porosity of about 10% and relative density around 90%, which is far below the value declared in the Markforged datasheet (above 97.6%).

The cubes from the TC1 set that have a nominal dimension equal to the FD samples, but were built with a 0.125 mm layer thickness, show relative density around 59% with residual internal porosity around 41%. Therefore, it could be assumed that the triangular infill strategy for the layer thickness equal to 0.125 mm produces an additional residual porosity of 31% when compared to the full-density strategy of the 0.50 mm layer thickness. However, for the bigger cubes of the TC2 set the residual porosity resulted in being even higher and around 45%. The differences in the final density could also explain the observed differences in the volumetric shrinkage. Hence a correlation between the layer thickness and the size of the sample may be envisaged.

### 3.2. Superficial Roughness Analysis

**Figure 6:**
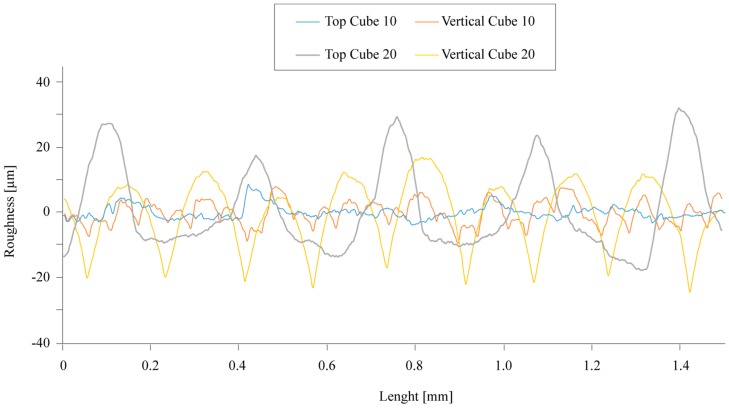
An example of roughness profiles for the cubic samples of sets TC1 and TC2.

Figure 6 shows the typical roughness profiles that were acquired on different surfaces of TC1 and TC2 cubes after sintering. The profiles are regular and periodic, as for other extrusion-based processes for which the surface roughness is largely affected by the filament deposition. The grooves are more evident for the vertical surfaces. Smaller cubes showed a lower surface roughness with an average value Ra of about 1.5 µm and 3.1 µm for the top and the vertical surfaces, respectively. The maximum value of 20.6 µm was measured for the roughness of the vertical surfaces. The bigger cubes are rougher. The Ra values are similar among the surfaces and measure around 9.5 µm and 9.1 µm for the top and the vertical surfaces, respectively. As for the smaller cubes, the maximum roughness of TC2 samples was detected for the vertical surfaces with a value Ra of 56.8 µm.

### 3.3. Analysis of the Dimensional Accuracy

**Figure 7:**
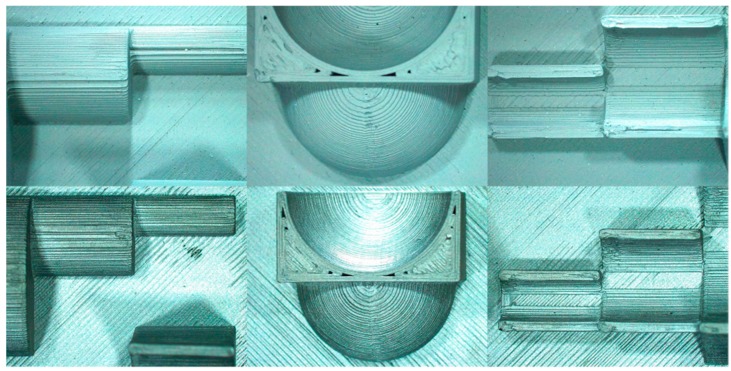
Images of the geometric features of the reference artefact for the green part (**above**) and the final sintered part (**below**).

The dimensional accuracy for the ADAM process is investigated for the sintered artefact only. Some images of the geometries of the reference part are shown in Figure 7 in the as-built state and final sintered one.

**Figure 8:**
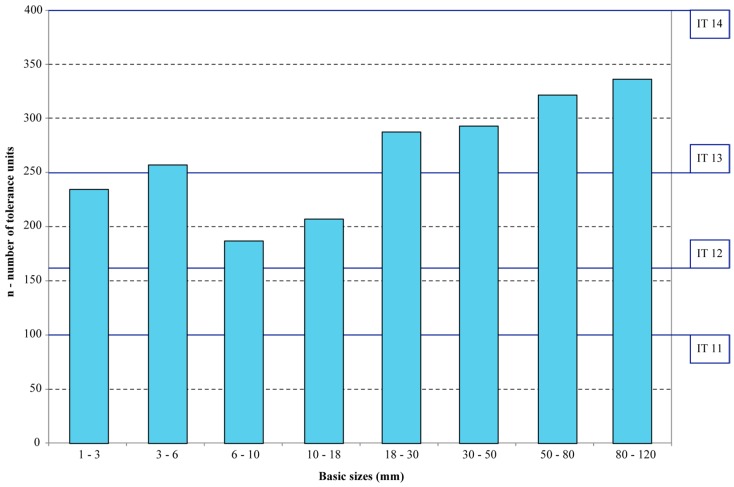
Dimensional accuracy (95th percentile) of the ADAM process in terms of IT grades for different ranges of ISO basic sizes.

The results of dimensional accuracy are reported in Figure 8 in terms of IT Grades. For the explored ISO ranges of basic size up to 120 mm, the artefact quality fits in the IT12 and IT13 classes. Unlike previous benchmarking results of extrusion-based AM systems for polymers [23,25], the accuracy of the ADAM process seems more constant over the ISO ranges. In layer-wise processes for polymers the dimensional quality is lower for features of small size, since an IT class higher than IT13 is normally achieved for the first two ISO ranges, from 1 to 3 mm and from 3 to 6 mm. For ISO sizes greater than 30 mm, the accuracy of AM systems for polymers generally fits into the IT11 or IT12 class. The increase in accuracy for larger sizes is not confirmed in Figure 8. The reason for this difference could be attributed to the sintering phase of the ADAM process. The application of an even oversizing factor of about 20% by Eiger software independently of the feature size results in the lowest dimensional accuracy at the end of the sintering phase for geometries of larger size and volume.

However, considering the presence of the sintering phase that involves a thermal treatment, the dimensional accuracy achieved by the ADAM process is consistent with that of other traditional processes for metals, like casting and forming [31]. Unlike polymers, that are injection moulded to their final shape and size, higher dimensional accuracy levels for metal components are achieved by finishing using metal cutting operations. In the same way, keeping into account the results in Figure 8, proper allowance for finishing operations should be assigned on the surfaces of parts to be produced by the ADAM process when the desired accuracy or roughness cannot be met by means of the layer-wise process only.

## 4. Conclusions

In this paper the results of analyses focusing on the density, surface roughness and dimensional accuracy of 17-4 PH samples manufactured by means of Atomic Diffusion Additive Manufacturing patented by Markforged Inc. are presented. This study aims at widening the knowledge about ADAM, a relatively new layer-wise process that has received little attention from a few papers only in the literature up to date.

The density analysis put in evidence that the ADAM process cannot achieve the same level of density of other AM processes that use a thermal source to locally melt metal powders. Depending on the adopted layer thickness between the two values allowed by the proprietary Eiger software, the parts are built with a full-density (FD) infill or a triangular cell (TC), one in the Metal X machine. Further post-processing operations are needed to debound the polymeric binder that encapsulates the metal powder in the filament and then sinter the green part into a furnace. When an FD infill is used, the maximum density achieved at the end of the sintering phase is about 90%, whereas half of this value is reached with the other infill strategy. This means that the voids left by the triangular empty cells cannot be filled during the last sintering step in the furnace.

Nevertheless, the dimensional accuracy of the final ADAM parts is rather good also for the TC infill and lower layer resolution. Markforged engineers have properly tuned the whole manufacturing workflow to provide parts with a precision similar to that of other traditional processes for metals like casting and forming. Further finishing operations should then be applied by machining processes to improve the part accuracy and the surface roughness.

Success case histories promoted by Markforged are related to the production of custom soft jaws for difficult machining operations or alignment jigs for welding. These kinds of products probably do not require the material to have a full density because the part is not subjected to high loads when it is put into operation. Therefore, the filament-based ADAM process is conceived for a viable economic alternative for the manufacturing of customised fixtures or jigs with easier material management than other AM alternatives that use metal powders.

Further research activities could be aimed at investigating the influence of different thermal treatments on the microstructure and final part resistance.

## Figures and Tables

**Table 1:** Composition of 17-4 PH material.

Cr	Ni	Cu	Si	Mn	Nb	C	P	S	Fe
15–17.5%	3–5%	3–5%	1% max	1% max	0.15–0.45%	0.07% max	0.04% max	0.03% max	bal

**Table 2:** Production data for the three sets of cubic samples.

Characteristic	Job A (Cubes FD)	Job B (Cubes TC1 and TC2)
Number of samples in the job	3 samples	5 samples for set TC1 5 samples for set TC2
Layer thickness [mm]	0.050	0.125
Total printing time [h]	10.12	26
Minimum washing time [h]	28	17
Printed mass for each cube [g]	16.05	13.94 (TC1) 50.54 (TC2)
Printed dimension for each cube [mm^3^]	12 × 12 × 12	12 × 12 × 12 (TC1) 23.9 × 23.9 × 23.9 (TC2)
Release ceramic for each cube [cm^3^]	0.01	0.02 (TC1) 0.08 (TC2)
Metal volume for each cube [cm^3^]	3.19	2.77 (TC1) 10.02 (TC2)

**Table 3:** Average dimensions with deviation range of the as-built samples and final dimensions of the cubes after sintering.

Sample	As-Built Cube			Sintered Cube		
	*w* [mm]	*l* [mm]	*h* [mm]	*w* [mm]	*l* [mm]	*h* [mm]
FD_1_	11.88 ± 0.01	11.96 ± 0.01	12.03 ± 0.01	9.88 ± 0.01	9.95 ± 0.01	10.09 ± 0.01
FD_2_	11.97 ± 0.01	11.96 ± 0.01	12.00 ± 0.01	9.96 ± 0.01	9.95 ± 0.01	10.04 ± 0.01
FD_3_	11.96 ± 0.02	11.95 ± 0.02	12.01 ± 0.02	9.94 ± 0.02	9.93 ± 0.02	10.07 ± 0.01
TC1_1_	12.03 ± 0.01	12.03 ± 0.02	12.13 ± 0.01	10.06 ± 0.08	10.02 ± 0.05	10.26 ± 0.01
TC1_2_	12.02 ± 0.02	12.01 ± 0.03	12.11 ± 0.01	10.04 ± 0.04	10.00 ± 0.05	10.29 ± 0.01
TC1_3_	11.99 ± 0.00	12.13 ± 0.01	12.13 ± 0.01	9.99 ± 0.02	10.08 ± 0.05	10.26 ± 0.00
TC1_4_	11.99 ± 0.00	12.11 ± 0.08	12.13 ± 0.00	9.99 ± 0.00	10.05 ± 0.12	10.26 ± 0.00
TC1_5_	12.03 ± 0.03	11.96 ± 0.02	12.13 ± 0.00	10.05 ± 0.07	9.96 ± 0.02	10.25 ± 0.00
TC2_1_	22.99 ± 0.01	23.13 ± 0.03	23.06 ± 0.01	19.97 ± 0.02	20.04 ± 0.11	20.15 ± 0.00
TC2_2_	23.05 ± 0.02	23.10 ± 0.08	23.06 ± 0.00	20.03 ± 0.02	20.02 ± 0.11	20.15 ± 0.01
TC2_3_	22.99 ± 0.06	23.13 ± 0.05	23.06 ± 0.01	19.97 ± 0.05	20.04 ± 0.09	20.16 ± 0.01
TC2_4_	22.98 ± 0.02	23.10 ± 0.06	23.06 ± 0.01	19.95 ± 0.06	20.02 ± 0.09	20.17 ± 0.00
TC2_5_	22.99 ± 0.05	22.95 ± 0.08	23.06 ± 0.01	20.01 ± 0.09	19.98 ± 0.04	20.15 ± 0.00

**Table 4:** Production data for the replica of the reference artefact.

Characteristic	Part A (Cubes P)	Part B Cubes TC1 and TC2)
Layer thickness [mm]	0.125	0.125
Nominal dimension [mm]	110.0 × 27.2 × 33.0	110.0 × 82.8 × 21.0
Total printing time [h]	22	38
Minimum washing time [h]	36	26
Printed mass [g]	264.27	478.8
Printed dimension [mm^3^]	131.5 × 32.5 × 39.4	131.5 × 99.0 × 25.1
Metal volume [cm^3^]	56.28	101.76

**Table 5:** Ranges of the International Organisation for Standardisation (ISO) basic sizes and corresponding tolerance factor *i*.

Range	Basic Size							
Above D_1_ (mm)	1	3	6	10	18	30	50	80
Up to including D_2_ (mm)	3	6	10	18	30	50	80	120
Standard tolerance factor *i* (µm)	0.542	0.733	0.898	1.083	1.307	1.561	1.856	2.173

**Table 6:** Classification of IT grades according to ISO 286-1:1988.

Range		IT 10	IT 11	IT 12	IT 13	IT 14	IT 15	IT 16
Above 1 mm	Up to 500 mm	64i	100i	160i	250i	400i	640i	1000i

**Table 7:** Density evaluation. The standard deviation for the weight in air and wet weight is below 0.0001 g, while for the weight in water is below 0.0400 g.

Cube	Weight in Air [g]	Weight in Water [g]	Wet Weight [g]	Effective Density [kg/m^3^]	Open Porosity [%]	Enclosed Porosity [%]	Relative Density [%]
FD_1_	6.8063	5.8466	6.8190	6978	8.6	10.0	90.0
FD_2_	6.8251	5.8725	6.8677	6837	7.5	11.8	88.2
FD_3_	6.8537	5.8880	6.8584	7042	8.7	9.1	90.9
TC1_1_	4.5569	3.5719	4.5647	4576	0.8	40.2	59.0
TC1_2_	4.5319	3.5445	4.5409	4535	0.9	40.6	58.5
TC1_3_	4.4848	3.4954	4.4980	4460	1.3	41.1	57.5
TC1_4_	4.4620	3.4816	4.4620	4538	0.0	41.4	58.6
TC1_5_	4.4730	3.4863	4.4745	4513	0.2	41.6	58.2
TC2_1_	27.7140	19.7867	27.7554	3467	0.5	54.7	44.7
TC2_2_	27.6968	19.7930	27.7201	3483	0.3	54.8	44.9
TC2_3_	27.7559	19.8627	27.8063	3483	0.6	54.4	45.0
TC2_4_	27.7311	19.9132	27.7764	3516	0.6	54.1	45.4
TC2_5_	27.6946	19.8484	27.7444	3496	0.6	54.2	45.1

**Table 8:** Density evaluation after the polishing. The standard deviation for the weight in air and wet weight is below 0.0001 g, while for the weight in water is below 0.0400 g.

Cube	Weight in Air [g]	Weight in Water [g]	Wet Weight [g]	Effective Density [kg/m^3^]	Open Porosity [%]	Enclosed POROSITY [%]	Relative Density [%]
FD_1_	6.8142	5.8402	6.8143	6974	0.0	10.0	90.0
FD_2_	6.79633	5.8245	6.7958	6976	0.0	10.0	90.0
FD_3_	6.8385	5.8669	6.8385	7017	0.0	9.4	90.5
TC1_1_	4.4730	3.5001	4.4727	4586	0.0	40.9	59.2
TC1_2_	4.4233	3.4475	4.4242	4516	0.1	41.6	58.3
TC1_3_	4.4104	3.4353	4.4104	4510	0.0	41.8	58.2
TC1_4_	4.4904	3.5136	4.4909	4581	0.0	40.8	59.1
TC1_5_	4.4105	3.4360	4.4112	4509	0.1	41.7	58.2
TC2_1_	27.3341	19.6654	27.5016	3478	2.1	53.0	44.9
TC2_2_	27.4985	19.6437	27.4981	3491	0.0	55.0	45.0
TC2_3_	27.4658	19.6172	27.4654	3489	0.0	55.0	45.0
TC2_4_	27.4687	19.6246	27.4689	3491	0.0	54.9	45.0
TC2_5_	27.4991	19.6468	27.4990	3492	0.0	54.9	45.1
